# Supplementary material for: Structures of the TMC-1 complex illuminate mechanosensory transduction
Source: Nature. 2022 Oct 12;610(7933):796–803. doi: 10.1038/s41586-022-05314-8 (PMC9605866; doi:10.1038/s41586-022-05314-8)
Supplement: Supplementary file 1 — This file contains Supplementary Figs. 1–3. [file 41586_2022_5314_MOESM1_ESM.pdf]

nature portfolio

https://doi.org/10.1038/s41586-022-05314-8

Supplementary information

Structures of the TMC-1 complex illuminate mechanosensory transduction

In the format provided by the authors and unedited

Structure of *C. elegans* TMC-1 complex illuminates auditory mechanosensory transduction

Hanbin Jeong[1*], Sarah Clark[1*], April Goehring[1,2], Sepehr Dehghani-Ghahnaviyeh[3], Ali Rasouli[3], Emad Tajkhorshid[3] and Eric Gouaux[1,2]

1. Vollum Institute, Oregon Health & Science University, Portland, Oregon 97239, USA.

2. Howard Hughes Medical Institute, Oregon Health & Science University, Portland, Oregon 97239, USA.

3. Theoretical and Computational Biophysics Group, NIH Center for Macromolecular Modeling and Bioinformatics, Beckman Institute for Advanced Science and Technology, Department of Biochemistry, and Center for Biophysics and Quantitative Biology, University of Illinois at Urbana-Champaign, Urbana, Illinois 61801, USA.

*These authors made equal contributions.

Correspondence to Eric Gouaux: gouauxe@ohsu.edu

This PDF file includes:

Supplementary Figures 1-3

Table of Contents	Title	Page
Supplementary Figure 1	*tmc-1::mVenus* strain	3
Supplementary Figure 2	N-terminal sequence of mouse TMIE	4
Supplementary Figure 3	Structural and sequence alignment of CALM-1, CIB2, and CIB3.	5

a

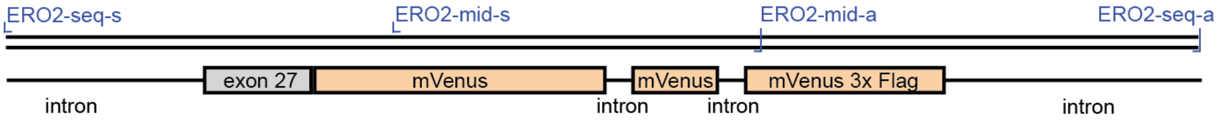

b syb2173 (*tmc1::mVenus*)

```
attagatcccgcaagagaatcgtcattctttcactataggttgtccattttcagtttttgacaggttttcgttattttta
aagttaaattgacccgttaaaaattagaactaaaataaattcaaaaataagtttaaatatcaaacatagcagtaatactta
aaaataaaacgaagagcgatattttcttcaaaattttttttgtattgtaattccccggattaccattgaagtatgctgac
agcaatgtgatgttcatctctatcaaaatagaataaaataatagtaaagttctagAGCTCAACATAAAATCGTATCACAAG
CATCGTCTAGCAGCTCAATTCCACATGGACGGCAACCGGATCCAAACAAGAAAGCGTCGCTTGTTTTGCCGCCGTTAAGAG
CCCCACGTGTTCAGTTTGATGAAGATGACTCTCCGCGGCAAATTGATGGCAGTGGTAGCCTCGAAGTACTCTTCCAAGGTC
CTGCCGCTGCGGCAGTAGTATCGAAAGGAGAGGAGTTGTTCACGGGTGTTGTCCCAATCCTCGTCGAGCTCGACGGAGACG
TCAACGGACACAAGTTCTCCGTCTCCGGAGAGGGAGAGGGAGACGCCACCTACGGAAAGCTCACCCTCAAGCTCATCTGCA
CCACCGGAAAGCTCCCAGTCCCATGGCCAACCCTCGTCACCACCCTCGGATACGGACTCCAATGCTTCGCCCGTTACCCAG
ACCACATGAAGCAACACGACTTCTTCAAGTCCGCCATGCCAGAGGGATACGTCCAAGAGCGTACCATCTTCTTCAAGgtaa
gtttaaacatatatatactaactaaccctgattatttaaattttcagGACGACGGAAACTACAAGACCCGTGCCGAGGTCA
AGTTCGAGGGAGACACCCTCGTCAACCGTATCGAGCTCAAGgtaagtttaaacagttcgtactaactaaccatacatattt
aaattttcagGGAATCGACTTCAAAGAAGACGGAAACATCCTCGGACACAAGCTCGAGTACAACTACAACTCCCACAACGT
CTACATCACCGCCGACAAGCAAAAGAACGGAATCAAGGCCAACTTCAAGgtaagtttaaacatgattttactaactaacta
atctgatttaaattttcagATCCGTCACAACATCGAAGATGGAGGAGTCCAACTCGCCGACCACTACCAACAAAACACCCC
AATCGGAGACGGACCAGTCCTCCTCCCAGACAACCACTACCTCTCCTACCAATCCAAGCTCTCCAAGGACCCAAACGAGAA
GCGTGACCACATGGTCCTCCTCGAGTTCGTCACCGCCGCCGGAATCACCCTCGGAATGGACGAGCTCTACAAGGGCAGTAC
CGGTAGCGACTACAAAGATCATGATGGCGATTACAAGGACCATGATATTGATTATAAGGATGATGACGATAAATGAttttt
ttttgtttttttcgaagaaatcttttgctccctccggtggacttgctcctggctgcaggaatatccggttgaaatatttaa
tctaattctagtttcatttcgtttttttctctttttttgccattttatttgtgaacattccatcgaaaaactcatttt
ctgaaacaaaaattcacaaaactctttctaactgatctctctgtttcttttcatatatttaactattttattcaatttct
catttttctgtcactactactctatttctaaaatacccgcaaagtaaggtacattaaatgatcttgaaccccttgcaaaac
cgttccattacgtttttccggaaacttttcctgtttcttctttccttttcaatcatttcggttcgttcatatcacctt
```

Supplementary Fig. 1. ***tmc-1::mVenus* strain. a**, Schematic diagram of *tmc-1::mVenus* strain. Location of the PCR and sequencing primers relative to the insertion site of 3C precision protease site-mVenus-3xFLAG prior to the stop codon of *tmc-1*in exon 27. **b,** Sequence of the *tmc-1::mVenus* strain. Introns are in lower case and exons in upper case letters. The 3C precision protease site-mVenus-3xFLAG insert is highlighted in orange. Primer annealing sites for PCR amplification and sequencing of insert are labeled with blue lines.

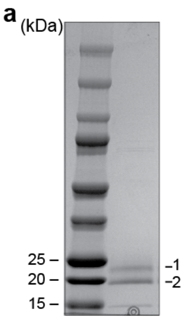

b

```
M. musculus    1   MAG-------RQHGSG-----RLWALGG---AALGACLAGVATQLVEPS--TAPPKPKPPPLTKETVVFW   53
H. sapiens     1   MA--------GWPGAG-----PLCVLGG---AALGVCLAGVAGQLVEPS--TAPPKPKPPPLTKETVVFW   52
D. rerio       1   MRRGRRRGKMAMEGPGNQPPSLVPLLMSVTAVLISHHFFNAAAQIPDPELLPTDPPKKPDPVTSETVVFW   70
C. elegans     1   MPSGNEE--------------------------INHL-SAL----------------------DQFVAP   20

M. musculus   54   DMRLWHVVGIFSLFVLSIIITLCCVFNCRVPRTRKEIEARYLQRKAAKMYTDKLETVPPLNELTEIPGED  123
H. sapiens    53   DMRLWHVVGIFSLFVLSIIITLCCVFNCRVPRTRKEIEARYLQRKAAKMYTDKLETVPPLNELTEVPGED  122
D. rerio      71   GLRLWQVVGIFSMFILAIIITLCCIFKCRIPRTKKEIEARHAQRLAAKKYANTLETVPPLNELTEVPGAA  140
C. elegans    21   GLRLWMLIALVGGVLLIMIVIVCCFMRIRIPRTKRQIDLIAAKRKLRKSTKNSAEANAH-----------   79

M. musculus  124   --KKKKKKDSVDTVAIKVEEDEKNEA-KKKGEK-------------------------------------  153
H. sapiens   123   KKKKKKKKDSVDTVAIKVEEDEKNEAKKKKGEK-------------------------------------  155
D. rerio     141   KVEVK---EEVPTVAGKVDGEKEKKKKEKESKKEGKGAKEGKEEEKEEPAKKKGEKGEKGEKGAPKKEAS  207
C. elegans    80   -------------------NDERAQAIVMNSMPSGGG------------------------GGAPSTSSS  106

M. musculus        ------------------------
H. sapiens         ------------------------
D. rerio     208   DGGKGKKGGEKAEEKGGAKKPAKK  231
C. elegans   107   -----RHTGSRIQSQV--------  117
```

Supplementary Fig. 2. N-terminal sequence of mouse TMIE. a, Representative Coomassie staining of sodium dodecyl sulfate-polyacrylamide gel electrophoresis of recombinantly expressed mouse TMIE. Upper and lower bands for mouse TMIE sample submitted for N-terminal sequencing and LC-MS/MS are indicated as 1 and 2, respectively. The experiments were repeated two more times with similar results. **b,** Results from N-terminal sequencing of mouse TMIE and mass spectrometry for both mouse and *C. elegans* TMIE. The cleavage site for both mouse TMIE bands was identical and is indicated with an arrow. The identified peptides for both mouse and *C. elegans* TMIE are underlined in red and the signal peptide and transmembrane domain are highlighted in grey and orange, respectively. The peptides that were identified for mouse TMIE were the same in both the upper and lower bands, suggesting the difference in the upper and lower bands is not due to N-terminal or C-terminal cleavage and is most likely due to post translational modification.

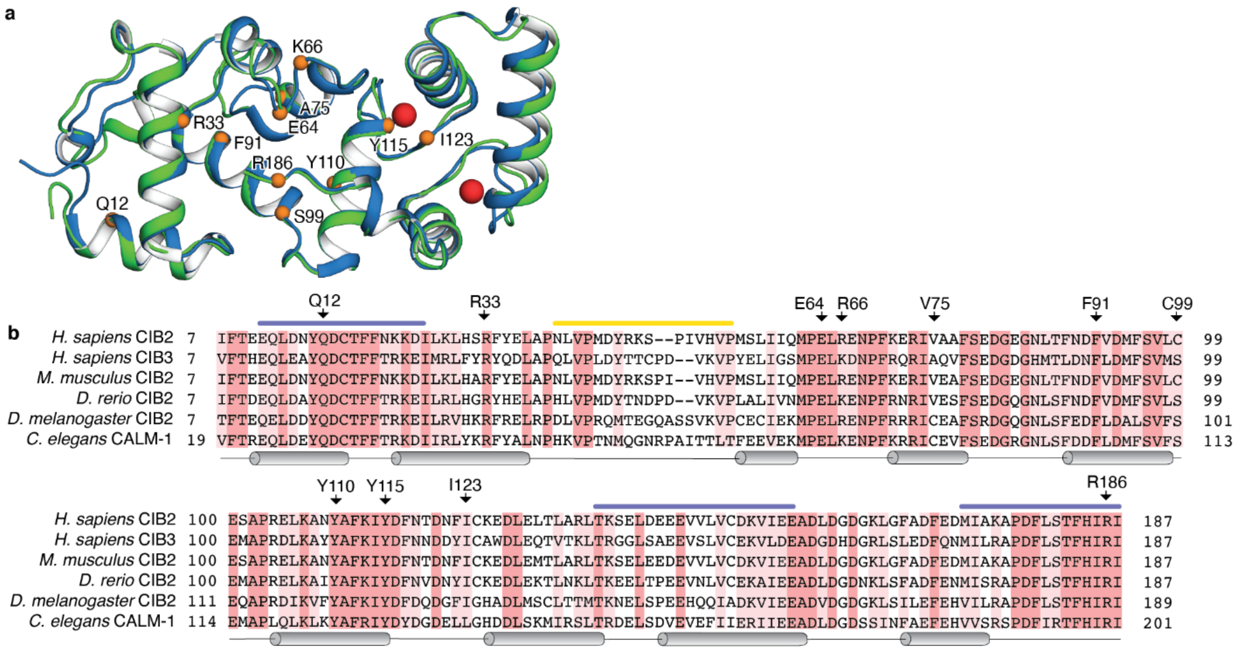

Supplementary Fig. 3. Structural and sequence alignment of CALM-1, CIB2, and CIB3.

a. Superposition of *C. elegans* CALM-1 (green) and human CIB3 (blue, PDB 6WUD) using backbone α - carbon atoms highlights structural conservation (RMSD = 0.7 Å). Calcium (CALM-1) and magnesium (CIB3) ions are shown as red spheres. CIB2 residues implicated in deafness or hearing loss mutations are shown as orange spheres. **b.** Sequence alignment of human CIB2, human CIB3, and CIB2 orthologs from mouse, zebrafish, fly, and worms. Identical residues are highlighted in red and similar residues are highlighted in pink. Regions involved in interactions with TMC-1 are depicted as blue bars and the region of ARRD-6 interaction is shown as a yellow bar. Secondary structure elements are shown below the sequence and the location of human CIB2 deafness mutations are indicated above the sequence.
