# Supplementary material for: Structures of the TMC-1 complex illuminate mechanosensory transduction
Source: Nature. 2022 Oct 12;610(7933):796–803. doi: 10.1038/s41586-022-05314-8 (PMC9605866; doi:10.1038/s41586-022-05314-8)
Supplement: Supplementary file 2 — Reporting Summary [file 41586_2022_5314_MOESM2_ESM.pdf]

nature portfolio

Corresponding author(s): Eric Gouaux

Last updated by author(s): Aug 16, 2022

Reporting Summary

Nature Portfolio wishes to improve the reproducibility of the work that we publish. This form provides structure for consistency and transparency in reporting. For further information on Nature Portfolio policies, see our Editorial Policies and the Editorial Policy Checklist.

Statistics

For all statistical analyses, confirm that the following items are present in the figure legend, table legend, main text, or Methods section.

n/a	Confirmed	
☐	☒	The exact sample size (*n*) for each experimental group/condition, given as a discrete number and unit of measurement
☐	☒	A statement on whether measurements were taken from distinct samples or whether the same sample was measured repeatedly
☒	☐	The statistical test(s) used AND whether they are one- or two-sided<br>*Only common tests should be described solely by name; describe more complex techniques in the Methods section.*
☒	☐	A description of all covariates tested
☐	☒	A description of any assumptions or corrections, such as tests of normality and adjustment for multiple comparisons
☐	☒	A full description of the statistical parameters including central tendency (e.g. means) or other basic estimates (e.g. regression coefficient) AND variation (e.g. standard deviation) or associated estimates of uncertainty (e.g. confidence intervals)
☒	☐	For null hypothesis testing, the test statistic (e.g. *F*, *t*, *r*) with confidence intervals, effect sizes, degrees of freedom and *P* value noted<br>*Give P values as exact values whenever suitable.*
☒	☐	For Bayesian analysis, information on the choice of priors and Markov chain Monte Carlo settings
☒	☐	For hierarchical and complex designs, identification of the appropriate level for tests and full reporting of outcomes
☒	☐	Estimates of effect sizes (e.g. Cohen's *d*, Pearson's *r*), indicating how they were calculated

Our web collection on statistics for biologists contains articles on many of the points above.

Software and code

Policy information about availability of computer code

Data collection	SerialEM 3.8
Data analysis	CryoSparc 3.3.1, PyMOL 2.4.1, Chimera 1.16, ChimeraX 1.1, MOLE 2.0, PHENIX 1.20, COOT 0.9, Alphafold2 (AF-D3KZG3-F1)

For manuscripts utilizing custom algorithms or software that are central to the research but not yet described in published literature, software must be made available to editors and reviewers. We strongly encourage code deposition in a community repository (e.g. GitHub). See the Nature Portfolio guidelines for submitting code & software for further information.

Data

Policy information about availability of data

All manuscripts must include a data availability statement. This statement should provide the following information, where applicable:

- Accession codes, unique identifiers, or web links for publicly available datasets
- A description of any restrictions on data availability
- For clinical datasets or third party data, please ensure that the statement adheres to our policy

WormBase gene code for TMC-1: T13G4.3.1. The coordinates and volumes for the cryo-EM data have been deposited in the Electron Microscopy Data Bank under accession codes EMD-26741 (Expanded), EMD-26742 (Contracted), and EMD-26743 (with ARRD-6). The coordinates have been deposited in the Protein Data Bank under accession codes 7USW (Expanded), 7USX (Contracted), and 7USY (with ARRD-6). All the initial and final snapshots of the MD trajectories, as well as simulation parameter and configuration files are deposited at https://doi.org/10.5281/zenodo.6780283.

Field-specific reporting

Please select the one below that is the best fit for your research. If you are not sure, read the appropriate sections before making your selection.

☒ Life sciences ☐ Behavioural & social sciences ☐ Ecological, evolutionary & environmental sciences

For a reference copy of the document with all sections, see nature.com/documents/nr-reporting-summary-flat.pdf

Life sciences study design

All studies must disclose on these points even when the disclosure is negative.

Sample size	Sample sizes were not predetermined for this study. Sample sizes of cryo-EM data were determined by the availability of the microscope time. Single molecule pulldown experiments were carried out by analyzing 200 spots each from three movies. For spectral confocal imaging, ten worms of different larval and adult stages were imaged in two separate experiments. The sample sizes of these both experiments were determined based on the consistency and variability.
Data exclusions	The following exclusions were pre-established. Particles were removed if their 3D reconstructions had poor quality.
Replication	All experiments were performed with independent replicates as described and replicates were successful within expected variation. Cryo-EM related experiments including protein purification and SDS-PAGE gels were successfully reproduced at least three times independently. Spectral confocal images of ten worms of different larval and adult stages were collected in two separate experiments, on different days, and yielded identical results. Single molecule pulldown experiments were successfully replicated two times independently.
Randomization	Randomization is not relevant to the biochemical and structural experiments described in this work and would not impact the results of the experiments.
Blinding	The investigators were not blinded. Blinding is not technically or practically feasible for the biochemical and structural experiments described in this work and would not impact the interpretation of the results.

Reporting for specific materials, systems and methods

We require information from authors about some types of materials, experimental systems and methods used in many studies. Here, indicate whether each material, system or method listed is relevant to your study. If you are not sure if a list item applies to your research, read the appropriate section before selecting a response.

Materials & experimental systems

n/a	Involved in the study	
☐	☒	Antibodies
☒	☐	Eukaryotic cell lines
☒	☐	Palaeontology and archaeology
☐	☒	Animals and other organisms
☒	☐	Human research participants
☒	☐	Clinical data
☒	☐	Dual use research of concern

Methods

n/a	Involved in the study	
☒	☐	ChIP-seq
☒	☐	Flow cytometry
☒	☐	MRI-based neuroimaging

Antibodies

Antibodies used	Commercial antibodies: Anti-Flag M2 Affinity Gel (Sigma, A2220, lot:SLCJ7861), Anti-GFP nanobody: (https://www.addgene.org/browse/article/6869/). GFP nanobody used for single molecule pulldown experiments was diluted to at a concentration of 1 to 3 µg/mL.
Validation	Validation of the anti-GFP nanobody used for single molecule pulldown experiments can be found in the published literature (PMID: 20945358). Validation for the Anti-FLAG M2 affinity gel can be found on the manufacturers website (https://www.sigmaaldrich.com/US/en/product/sigma/a2220).

Animals and other organisms

Policy information about studies involving animals; ARRIVE guidelines recommended for reporting animal research

Laboratory animals	Transgenic C. elegans were generated by SunyBiotech (strain: PHX2173 tmc-1(syb2173)). Worms of mixed larval and adult ages were grown and maintained in an incubator set at 20C.
Wild animals	The study did not involve wild animals.

Field-collected samples	No field-collected samples were used in this study.
Ethics oversight	C. elegans experiments do not require ethical approval.

Note that full information on the approval of the study protocol must also be provided in the manuscript.
