# Supplementary material for: Structures of the TMC-1 complex illuminate mechanosensory transduction
Source: Nature. 2022 Oct 12;610(7933):796–803. doi: 10.1038/s41586-022-05314-8 (PMC9605866; doi:10.1038/s41586-022-05314-8)
Supplement: Supplementary file 4 — Raw data for original silver-stained and Coomassie-stained gels. a, Original silver-stained gel (see Extended Data Fig. 1b (inset)). b, Original Coomassie-stained gel (see Supplementary Fig. 2a). Yellow boxes indicate how the gels were cropped. [file 41586_2022_5314_MOESM4_ESM.pdf]

a

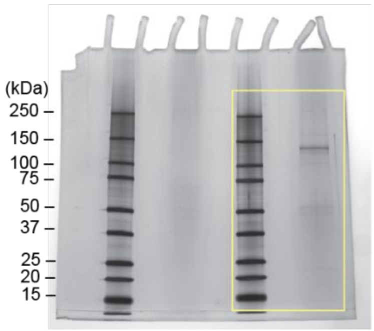

b

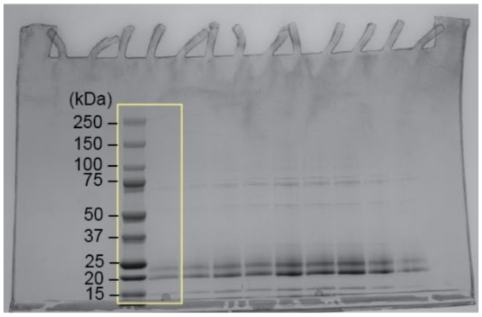

Raw data for original silver-stained and Coomassie-stained gels. a, Original silver-stained gel (see Extended Data Fig.1b (inset)). **b,** Original Coomassie stained gel (see Supplementary Fig. 2a). Yellow boxes indicate how the gels were cropped.
